# High catalytic activity of oriented 2.0.0 copper(I) oxide grown on graphene film

**DOI:** 10.1038/ncomms9561

**Published:** 2015-10-16

**Authors:** Ana Primo, Ivan Esteve-Adell, Juan F. Blandez, Amarajothi Dhakshinamoorthy, Mercedes Álvaro, Natalia Candu, Simona M. Coman, Vasile I. Parvulescu, Hermenegildo García

**Affiliations:** 1 Departamento de Quimica, Instituto Universitario de Tecnología Química (CSIC-UPV), Avenida de los Naranjos S/N, 46022 Valencia, Spain; 2 School of Chemistry, Madurai Kamaraj University, Madurai 625 021, Tamil Nadu, India; 3 Department of Organic Chemistry, Biochemistry and Catalysis, University of Bucharest, Bulevardu Regina Elisabeta nr. 4-12, 030018 Bucharest, Romania

## Abstract

Metal oxide nanoparticles supported on graphene exhibit high catalytic activity for oxidation, reduction and coupling reactions. Here we show that pyrolysis at 900 °C under inert atmosphere of copper(II) nitrate embedded in chitosan films affords 1.1.1 facet-oriented copper nanoplatelets supported on few-layered graphene. Oriented (1.1.1) copper nanoplatelets on graphene undergo spontaneous oxidation to render oriented (2.0.0) copper(I) oxide nanoplatelets on few-layered graphene. These films containing oriented copper(I) oxide exhibit as catalyst turnover numbers that can be three orders of magnitude higher for the Ullmann-type coupling, dehydrogenative coupling of dimethylphenylsilane with *n*-butanol and C–N cross-coupling than those of analogous unoriented graphene-supported copper(I) oxide nanoplatelets.

## Abstract (web summary)

Supported metal nanoparticles have been widely used as heterogeneous catalysts. Here, the authors report the synthesis of (1.1.1) copper on few layer graphene which oxidize to orientated (2.0.0) copper(I) oxide nanoplatelets which display high catalytic activity for a number of organic coupling reactions.

## Graphical abstract

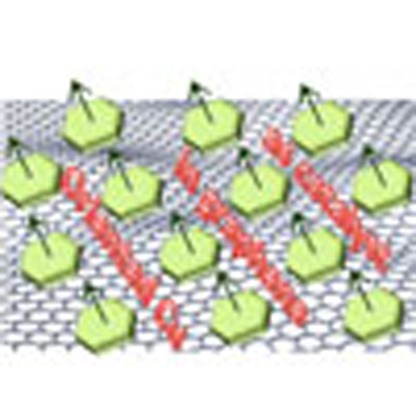

Metal nanoparticles (MNPs) supported on large surface area solids are widely used as heterogeneous catalysts for a large variety of organic reactions including reductions^1^,^2^,^3^,^4^, oxidations^5^,^6^,^7^ and couplings^8^,^9^,^10^ among other processes. One of the key issues in this area is how to control the preferential facets of the MNPs exposed to the reaction, since theoretical calculations as well as experimental data suggest that different crystallographic planes may exhibit specific activity in catalysis^11^. Among the numerous types of large area materials that have been used as supports, there are abundant data in the literature showing that graphene (G) as support of MNPs exhibits unique features not found in other solid supports^12^,^13^ that make these graphene-based composites containing MNPs as suitable catalysts^14^ and photocatalysts^15^,^16^. G being a one atom thick layer of *sp*^2^ carbons in hexagonal arrangement constitutes the physical limit of two-dimensional materials in which all constitutive atoms are accessible to interact with substrates and reagents. Theoretical calculations predict that the overlap between the extended *π* orbital of G and the *d* orbitals of transition metals may result in a strong metal-support interaction that can modulate the electronic density at the MNPs-G interface^17^,^18^,^19^,^20^. In addition, the large surface area of G and its high adsorption capacity should cooperate to the reaction mechanism by adsorbing substrates near the active MNP sites. Both properties of G, its ability to interact with MNPs and its high adsorption capacity, are highly desirable to act as support for MNPs. In a related precedent to the present work, the formation of powders of 1.1.1 oriented CuPd nanoplatelets (NPs) on G has been reported starting from (Cu^2+^–Pd^2+^)-containing graphene oxide that was reduced at room temperature with NaBH_4_ (ref. 21). The resulting oriented CuPd/G was used as catalyst for Pd-catalysed transformation of glycerol into lactic acid^21^. The same group has also reported the prior preparation of oriented 1.1.0 Cu_2_O NPs using sodium dodecylsulfate as template and subsequent adsorption of these preformed oriented Cu_2_O NPs on graphene oxide^22^.

Herein, we go forward in this direction showing a one-step synthesis of oriented (1.1.1) Cu NPs on few-layered graphene (*fl−*G) films as well as their selective oxidation to Cu(I) that becomes also facet oriented in the (2.0.0) plane. The key issue is the oriented growth of Cu NPs as G starts to develop in the one-step synthesis of the G-supported Cu NPs. Activity data show that as consequence of this preferential orientation these Cu_2_O-containing few-layers (*fl*) G films exhibit a remarkably high catalytic activity compared with related Cu catalysts with random crystalline orientation of Cu_2_O NPs for three coupling reactions.

## Results

### Synthesis of 
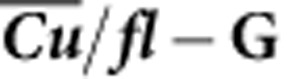


Our preparation procedure for oriented Cu NPs on top of *fl−*G films (
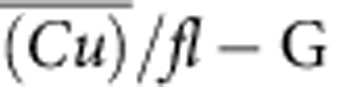
, 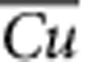 meaning 1.1.1 oriented Cu NPs) is inspired in the procedure of preparation of high electronic quality G films by chemical vapour deposition^23^,^24^,^25^. In this procedure of G preparation, a clean Cu or Ni metallic surface is used to grow G films by pyrolysis at temperatures about 1,500 °C employing methane as carbon source in hydrogen atmosphere. It is accepted that under these conditions, methane is decomposed into hydrogen and carbon atoms that start to deposit on the metal surface^23^,^26^. The low solubility of C in Cu makes this metal especially suited for the preparation of G, since metal carbides are not formed^27^,^28^,^29^. Up to six carbon atoms can fit around a single metal atoms. As result, the atomically flat metal surface acts as template in the formation of G and each hexagon is formed crowning a metal atom of the surface. In chemical vapour deposition (CVD) studies, it has been proved that the 1.1.1 facet of Cu films is more suited to form high-quality G compared with the 1.0.0 facet that matches worse with the symmetry and dimensions of G (refs 30, 31, 32). Our leading hypothesis is that the same principles should also apply to the reverse process and G sheets formed spontaneously in the pyrolysis of natural polysaccharides could drive the growth of Cu NPs to a preferential crystallographic plane.

In the present case, preparation of films was performed by pyrolysis of Cu^2+^-chitosan films supported on quartz as substrate (see experimental procedure for the preparation of Cu^2+^-chitosan). Previously, we have found that films of *fl−*G can be obtained by pyrolysis under inert atmosphere and temperature about 1,000 °C of films of few nanometres thickness of chitosan and other filmogenic natural biopolymers^33^,^34^,^35^. Chitosan is able to form continuous, crack-free nanometric films with subnanometric roughness on arbitrary substrates. Owing to the tendency of polysaccharides to render graphitic carbon residues, subsequent pyrolysis of these chitosan films renders G or *fl−*G films conformal to the substrate. In the present case, it was anticipated that, on pyrolysis, Cu^2+^-containing chitosan films will form Cu NPs on G due to the spontaneous segregation of the two components during the pyrolysis. Chitosan and other polysaccharides are well known agents to trap metal ions in aqueous solution, including Cu^2+^, and for this reason they are widely employed in water purification^36^,^37^,^38^. On the other hand, the low solubility of Cu and C and the lack of formation of the corresponding metal carbide will determine that Cu atoms present in the Cu^2+^-chitosan film will segregate in a different phase as they become reduced to Cu NPs. In a prior study, pyrolysis of hybrid Ni^2+^–Mn^4+^ hydrotalcite/alginate solids led to a spontaneous carbochemical reduction of Ni^2+^ metal ions resulting in the segregation of Ni NPs on the carbon residue^39^. In view of these precedents, we anticipated that nanometric films of chitosan embedding Cu^2+^ could lead on pyrolysis to G-supported Cu NPs. Also it was expected that in the process, *fl−*G film could act as template determining the preferential growth of a certain facet of Cu NPs as they are formed on G. Experimental evidence supports that our hypotheses were in general terms correct.

**Figure 1:**
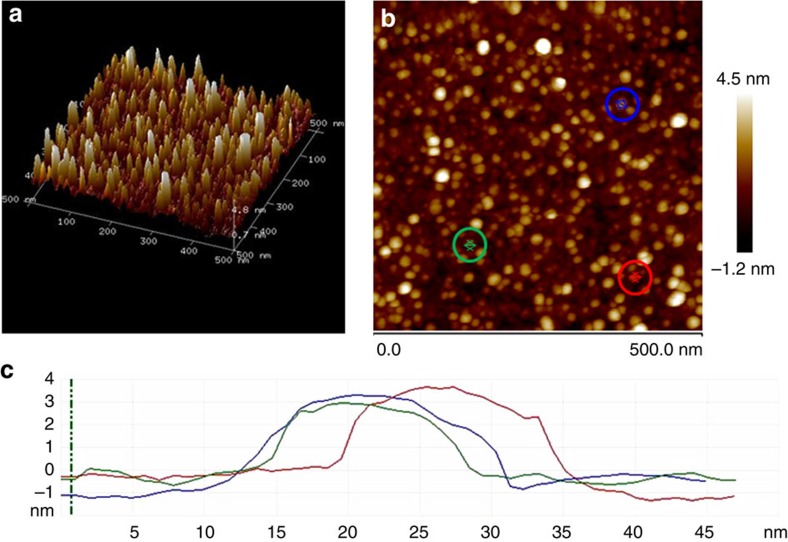
AFM of 
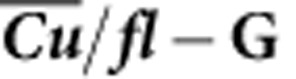
. General (0.5 × 0.5 μm) three-dimensional (**a**) and two-dimensional (**b**) views of the 
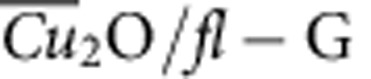
 films based on AFM. The height of three representative Cu NPs on G marked on blue, green and red (**c**) is presented in the bottom part with coincident colours.

Figure 1 shows the atomic force microscopy (AFM) images of the resulting 
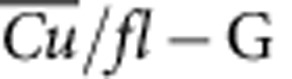
 obtained after pyrolysis.

One of the key points in the properties of the resulting 
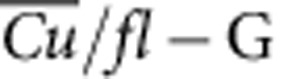
 is the high quality of the Cu^2+^-chitosan film of nanometric thickness (filmogenecity). In these nanometric Cu^2+^-chitosan films the presence of Cu^2+^ cannot be distinguished by electron microscopy or AFM, since Cu^2+^ ions should be highly dispersed, probably as individual ions, on the polysaccharide matrix by interaction with the amino groups of the polymeric fibrils. The presence of Cu^2+^ on chitosan can be, however, ascertained by analytical tools such as inductively coupled plasma optical emission spectrometry (ICP-OES) or X-ray photoelectron spectroscopy (XPS). Pyrolysis of chitosan films embedding Cu^2+^ ions results in a shrinking to less than one half of the initial film thickness as consequence of the formation and stacking of G sheets in the pyrolysis. According to these measurements, the 
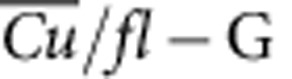
 is constituted by about 10 G sheets (4 nm height). On top of these G sheets, the presence of Cu particles can be observed (see Fig. 1). The subnanometric vertical resolution of the AFM equipment shows that Cu particles are flat and have a morphology as nanoplatelets, with an average high of about 3 nm and the frontal view shows that these thin Cu plates are homogenously distributed on top of the *fl−*G film.

**Figure 2:**
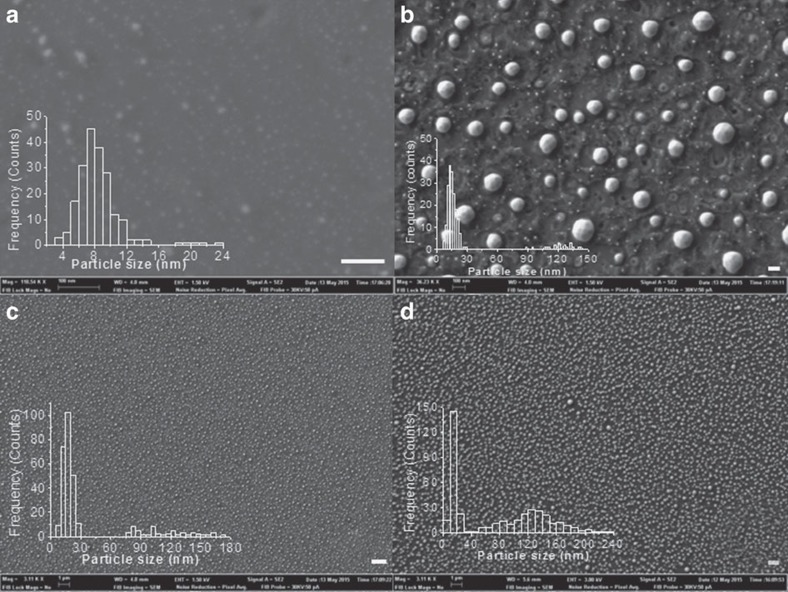
FESEM images of oriented Cu on few or multi layers G. FESEM images of 
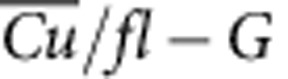
 (**a**,**c**) and 
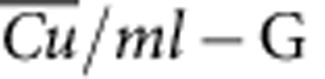
 (**b**,**d**) films at high (**a**,**b**) and low (**c**,**d**) magnification, showing the homogeneous distribution of Cu NPs over the G film. The insets in the panels show the statistical particle size distribution determined for each of the images of 
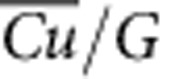
. Scale bars: **a**,**b**, 100 nm; **c**,**d**, 1 μm.

**Figure 3:**
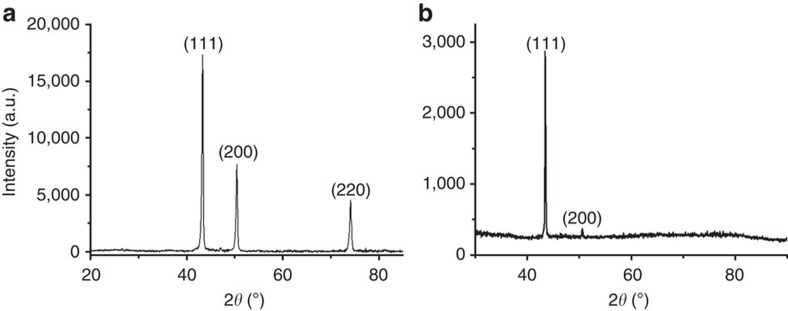
XRD patterns of oriented and unoriented Cu NPs supported on *ml*-G. (**a**) XRD of Cu NPs obtained by the polyol method; (**b**) XRD pattern for the pyrolysed Cu^2+^-chitosan films corresponding to 
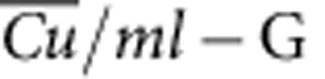
. Cu/*ml*–G is the sample show in Fig. 2b,d.

Field emission scanning electron microscopy (FESEM) images of 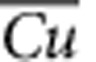-films are presented in Fig. 2. In agreement with AFM images, FESEM also confirms the high regularity of the morphology of Cu NPs, their uniform distribution and relatively small lateral particle size (from 5 to 20, average 8 nm, insets of Fig. 2), particularly considering the high preparation temperature (1,000 °C). FESEM images for 
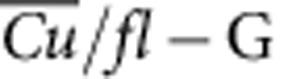
 suggest a high crystallinity and ordering of the metal atoms in the Cu nanoplatelets. Unfortunately, the nanometric films corresponding to Figs 1 and 2 (panels a and b) do not exhibit X-ray diffraction (XRD), due to the low thickness and Cu content and size in these films. The regularity of Cu nanoplatelets and the type of crystallographic facet preferentially grown in the Cu NPs obtained by this procedure were determined indirectly by analysing the XRD pattern of analogous samples obtained by pyrolysis of chitosan embedding Cu^2+^ as thicker films. Pyrolysis of thicker Cu^2+^-chitosan films (50 nm thickness in comparison with 10 nm for 
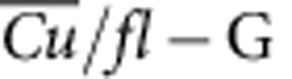
) render 
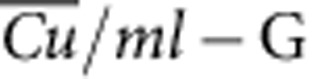
 samples (*ml*: multi layer) with sufficiently high Cu content to allow recording XRD. Figure 3 shows a XRD pattern obtained for pyrolysed Cu^2+^-chitosan films (50 nm thickness) as compared with the pattern of unoriented Cu NPs obtained by the polyol method^40^.

As it can be seen in Fig. 3, XRD of the pyrolysed Cu^2+^-chitosan films exhibits almost exclusively a single peak corresponding to the 1.1.1 plane in contrast to the XRD of conventional Cu NPs prepared by the polyol method that can be indexed undisputedly to cubic Cu (JCPDS no. 04-0836). The XRD recorded for thick Cu/*ml*–G films constitutes firm evidence that Cu NPs exhibit oriented facets when grown following the chitosan pyrolysis method.

**Figure 4:**
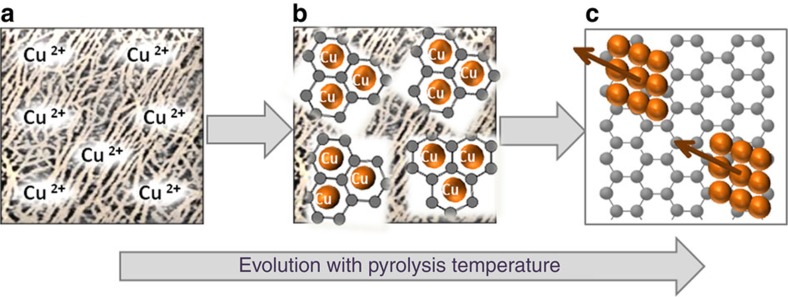
Mechanism of 
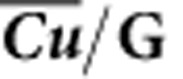
 formation. Proposal illustrating how the presence of a G sheet could template the preferential growth of Cu particles along the 1.1.1 facet. Frame (**a**) represents individual Cu^2+^ ions interacting with chitosan fibrils. Frame (**b**) illustrates an intermediate stage in which graphene sheet is being formed synchronously with some Cu^2^^+^ reduction to Cu(0) that is accommodated within the hexagonal arrangement of G. Frame (**c**) indicates how the growth of Cu nanoplatelets is templated by G sheet.

We propose that the preferential growth of this crystal plane is due to the epitaxial growth of Cu NPs templated by evolving G sheets, in a similar way as the proposed mechanism to explain the formation of G by CVD on Cu films already commented in the introduction. Figure 4 summarizes our proposal. In support of this proposal, the evolution of Cu particles and G as a function of the pyrolysis temperature was followed by FESEM (see Supplementary Fig. 1). It was observed that *fl*−G starts to develop at temperatures about 800 °C. At lower temperatures, no significant conductivity characteristic of G was observed and FESEM images were not possible or were of low quality. At 800 °C the presence of some Cu particles is already observed, although their number and density on the surface are very low compared with the number of nanoplatelets formed at 1,000 °C. The low density of Cu particles observed at 800 °C could be probably due to the fact that most Cu^2+^ ions are still not reduced and are mostly embedded on the amorphous carbon evolving from chitosan fibrils. From 800 to 900 °C, the degree of graphitization increases significantly as already reported and also the number of Cu particles that segregates increases considerably as the FESEM images show. The process seems to be complete at 1,000 °C. Thus, according to these images evolution of Cu nanoplatelets and G formation are relatively synchronous, allowing the preferential growth of Cu facets due to templation by G. An additional point to consider with respect to Cu–G interaction is the relatively small particle size of Cu NPs taking into account the high pyrolysis temperature (1,000 °C) and time (over 6 h) to which the samples are submitted. This relatively small Cu particle size suggests a certain control of particle growth by metal-support interaction as claimed in precedents in where an unexpectedly small particle size of metal oxides in the pyrolysis of precursors has been observed^41^.

**Figure 5:**
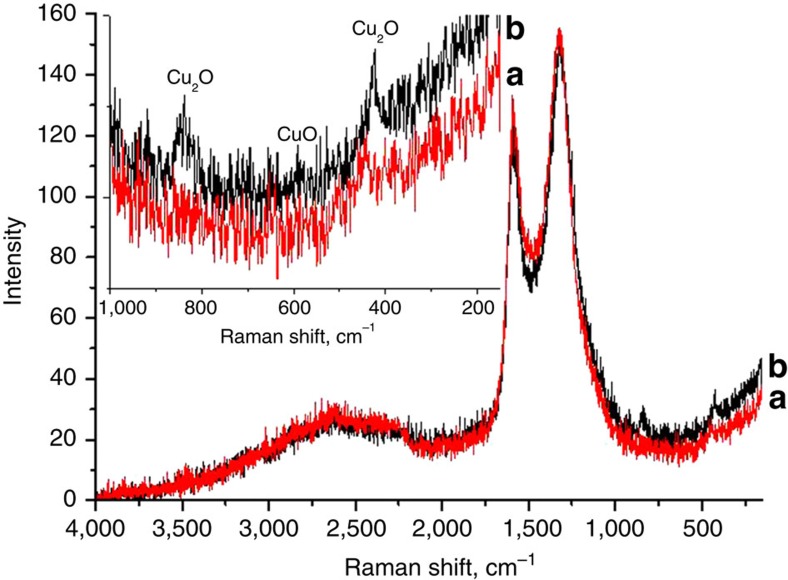
Raman spectroscopy of 
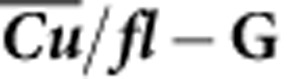
 films. Raman spectra of 
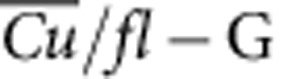
 films supported in quartz substrates (2 × 2 cm^2^) immediately before (**a**) using them as catalyst for the Ullmann-type coupling of iodobenzene and after (**b**) being used in the reaction. The inset shows and expansion of the low wavenumber region that allows distinguishing specific vibrational peaks for Cu(I) and Cu(II). Comparison of the two spectra show that while the bands corresponding to G undergo minor alterations under the reaction conditions, the peaks due to Cu species disappear from the quartz as consequence of 
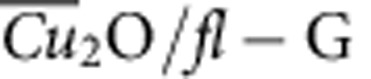
 detachment from the quartz plate (see text for explanation).

XRD of freshly prepared thick 
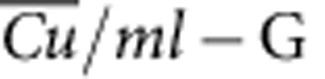
 samples correspond cubic Cu metal (JCPDS no. 04-0836). It is, however, frequently observed that Cu NPs undergo spontaneous oxidation on storage at the ambient. In the present case, the partial oxidation of 
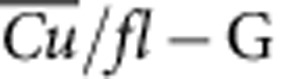
 films in contact with the atmosphere was determined by Raman spectroscopy. Figure 5 shows the Raman spectra of originally 
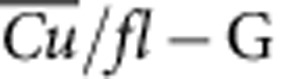
 film that has been stored at the ambient and before its use as catalyst. Raman spectroscopy of the ambient-exposed 
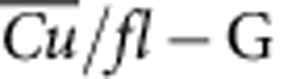
 films allows detecting together with the G additional peaks at 850, 435 and 180 cm^−1^ attributable to the presence of Cu_2_O based on abundant literature data^42^,^43^. It should be commented that Cu(0) cannot be detected by Raman spectroscopy.

**Figure 6:**
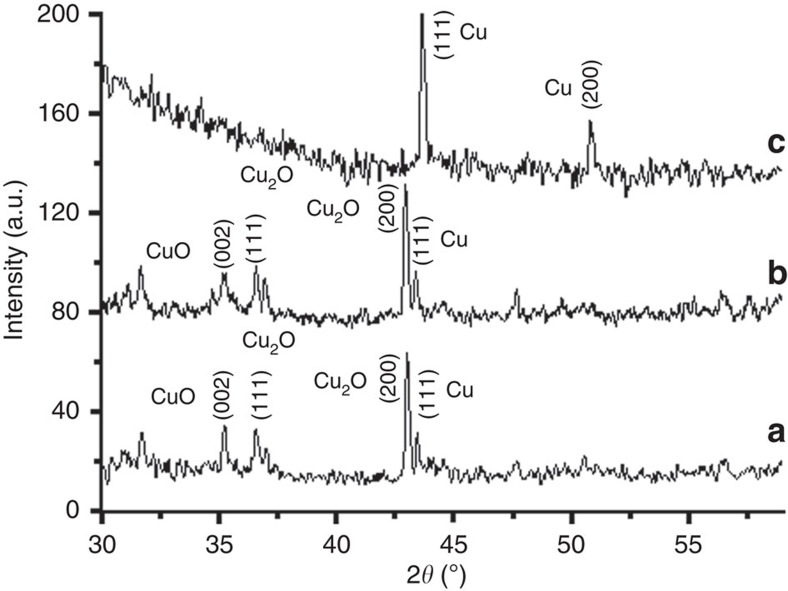
Variation of XRD as a function of the pretreatment. XRD spectra of 
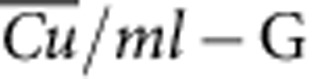
 exposed to the air resulting in 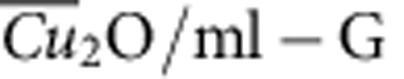 (**a**) and subsequent annealing under air at 300 °C for 1 h (**b**), followed by H_2_ reduction at 200 °C (**c**).

This spontaneous oxidation of Cu(0) also occurs for 
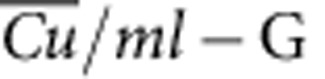
 for which the oxidation process can be followed by XRD (Fig. 6). It was observed that on oxidation of 
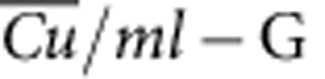
, the XRD of the film shows the formation of oriented 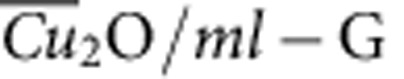 as determined by the relative intensity of the 2.0.0 peak that is much higher than that of the 1.1.1 facet. The presence of small peaks corresponding to CuO was also detected, but their intensity was much smaller than those of oriented Cu_2_O (see Fig. 6). Even an oxidation treatment consisting in heating at 300 °C under air flow for 1 h is not able to promote the complete oxidation of Cu(I) to Cu(II). One important observation was not only that Cu_2_O NPs were also oriented as the Cu NPs, but also that the process is reversible and reduction treatment by hydrogen restores from 2.0.0 oriented Cu_2_O the original Cu(0) NPs with preferential orientation as the initially formed NPs (Fig. 6 plot **c**). This cycling oxidation/reduction starting from oriented 
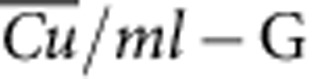
 was performed twice observing that preferential orientation for the 1.1.1 facet of Cu(0) NPs and 2.0.0 for Cu_2_O occurs reversibly in a large extent.

Thus, the information from vibrational spectroscopy for 
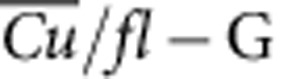
 together with the XRD for 
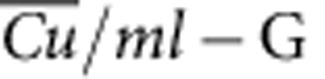
 confirm the changes in the oxidation state of Cu and the presence of oriented (1.0.0) Cu and (2.0.0) Cu_2_O NPs.

**Figure 7:**
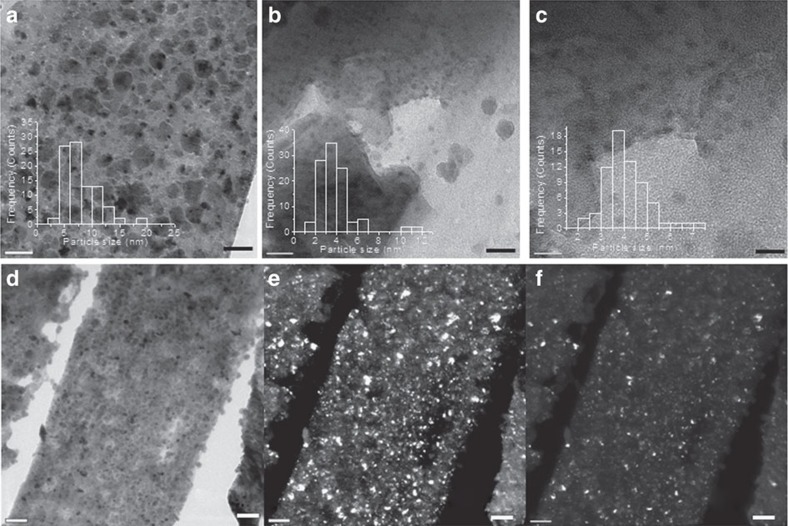
TEM images of 
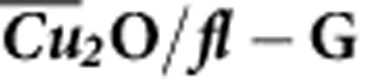
 detached from the quartz plate. (**a**,**b**,**c**) Set of TEM images of three different regions and various magnifications recorded for 
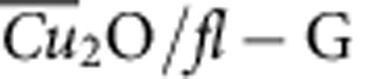
 after removal of quartz substrate showing the nanoplatelet morphology of Cu_2_O particles. The insets present the particle size distribution for each image with averages between 3.5 and 6 nm. Overall (**d**) and filtered for 2.0.0 (**e**) and 1.1.1 (**f**) facet orientation TEM images taken for strips of 
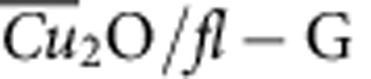
 sample after removal of quartz substrate. Particle counting indicates that 82% of the particles present in image **d** are also present in image **e**, indicating a preferential 2.0.0 facet orientation of Cu_2_O nanoplatelets. Scale bars, **a**, 50 nm; **b**, 20 nm; **c**, 10 nm; **d**,**e**,**f**, 0.1 μm.

To provide direct evidence of any preferential morphology and facet orientation of Cu_2_O particles, Cu_2_O/*fl*−G films were studied directly by transmission electron microscopy (TEM). TEM imaging requires the prior detachment of Cu_2_O/*fl*−G from the quartz substrate without disturbing orientation of the particles, a process that is not obvious. To avoid any possible influence on the orientation of Cu_2_O particles supported on *fl−*G in the detachment from quartz, the substrate was submitted to consecutive mechanical polishing up to 100 μm thickness, dimpling grinding and argon ion milling until complete removal of quartz^44^. The resulting self-standing 
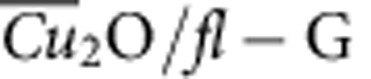
 film was introduced directly in the TEM chamber without deposition on a holley copper grid. The images a–c presented in Fig. 7 and Supplementary Fig. 2 revealed that the 
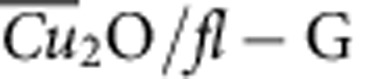
 sample is constituted also by nanoplatelets as in the case of 
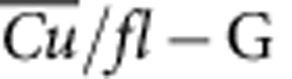
. In addition, these Cu_2_O nanoplatelets present a preferential 2.0.0 facet orientation as in the case of thick 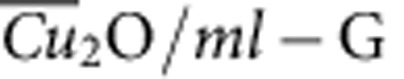 films determined by XRD. Figure 7 shows three representative images to illustrate the preferential 2.0.0 facet orientation of 
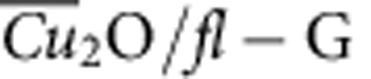
 as deduced by TEM. In panel d of Fig. 7 an image of all Cu_2_O nanoplatelets is presented and the information of this image is treated based on the selective area electron diffraction pattern to present in panel e exclusively those nanoplatelets that have 2.0.0 orientation. An analogous treatment of the raw TEM image to show nanoplatelets oriented in the 1.1.1 facet is shown in panel f of Fig. 7. Comparison of images e and f in Fig. 7 provides visual evidence that most of the Cu_2_O nanoplatelets exhibit the 2.0.0 facet. Counting a statistically relevant number of particles by means of ImageJ, an imaging program used for image comparison, allows estimating that about 82% of them have 2.0.0 facet orientation. It should be mentioned that the real percentage of oriented nanoplatelets could be even higher, since the necessary quartz removal treatment and loss of planarity may result in an apparent decrease of the number of oriented platelets.

**Figure 8:**
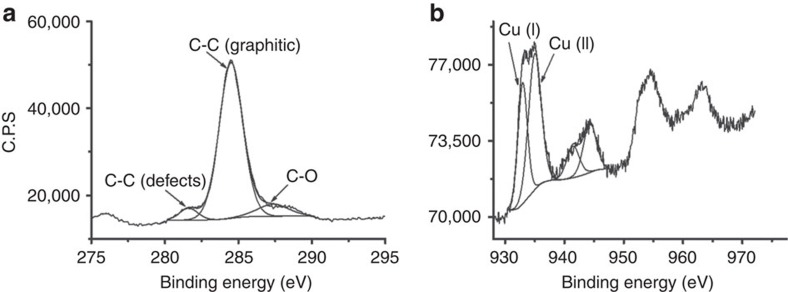
High-resolution XPS measurements. XPS peaks recorded for 
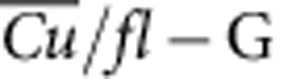
 showing the experimental C1s (**a**) and the Cu2p (**b**) and their best deconvolution to individual components, as indicated in the panels.

XPS also shows the presence of Cu(I) and Cu(II) on the outermost layers of oriented 
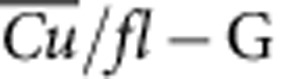
 and 
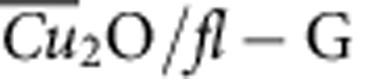
 films (Fig. 8). The shallow penetration depth of XPS allows probing exclusively the outermost layers of the nanoplatelets and this technique in combination with the Auger peak reveals that the external layers of the nanoplatelets are constituted by Cu(I) and Cu(II), based on the observation of a major component at a binding energy of 933 eV contributing in a 0.3 atom%, together with the component corresponding to Cu(II) at 935 eV contributing in a 0.8 atom%. It should be noted that this atomic proportion corresponds to the shallow region probed by XPS. Importantly, the influence of the presence of Cu on G is revealed by the fact that, even though chitosan contains N and it has been reported that its pyrolysis renders N-doped G (refs 33, 34), in the present case no peak corresponding to residual N is observed in the XPS. Moreover, the C1s peak in notably narrow centred at 284.5 eV, with components due to C atoms with dangling bonds at holes and a residual, minor population of C–O (<5 atom%), indicating that the quality of G is high. Thus, apparently, the presence of Cu nanoplatelets also influences G as support healing defects by removing completely N doping atoms from G and leaving a minor proportion of oxygenated functional groups.

### Catalytic activity

As commented in the introduction, one of the main applications of MNPs supported on G is their use as catalysts. The ability to prepare oriented Cu_2_O nanoplatelets allows us to test the influence of facet orientation on the activity for certain reactions catalysed by Cu(I). To illustrate how preferential orientation can influence the catalytic activity, three different reaction types characteristic of Cu(I) sites, namely: (i) the Ullmann self coupling of iodobenzene (Eq. 1), (ii) the dehydrogenative coupling of dimethylphenylsilane with *n*-butanol to form the corresponding *n-*butoxysilane (Eq. 2), and (iii) the C–N coupling of aniline and bromobenzene (Eq. 3) were selected.

A classical reaction catalysed by Cu(I) is the Ullmann coupling of aryl halides. To gain information about the specific activity of the 2.0.0 facets of Cu(I), the catalytic performance of 
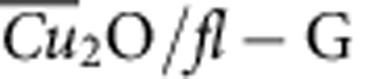
 films coating quartz substrates was checked for the Ullmann-type self coupling of iodobenzene (**1**) (refs 45, 46, 47). The amount of Cu in 
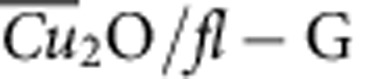
 determined by inductively coupled plasma optical emission spectrometry analysis was 0.23±0.05 μg of Cu per 1 × 1 cm^2^ plate. The activity of these oriented 
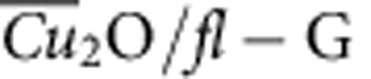
 films was compared with that of a synthesized Cu_2_O NPs (average particle size 5–7 nm) obtained by the ambient oxidation of Cu NPs prepared by the polyol method that have been supported on *fl−*G (Cu_2_O/*fl−*G). There are numerous reports in the literature showing that reduction of transition metals by thermal treatment in ethylene glycol renders MNPs of homogeneous particle size around 5 nm (refs 48, 49, 50, 51, 52). This polyol method to obtain unoriented Cu NPs has been already employed for the preparation of MNPs supported on G and other carbon nanoforms and used as heterogeneous catalyst^53^. In the present case, Cu NPs supported on *fl−*G were prepared at 1 and 0.1 wt% loading. Characterization data of the *fl−*G sample used as support that is coincident with that previously reported in the literature is provided in Supplementary Fig. 3 (ref. 34). Importantly XRD of the as-prepared Cu/*fl−*G sample shows the presence of Cu without any preferential facet exposed (see Fig. 3a). Spontaneous oxidation of this sample by exposure to the ambient renders again Cu_2_O/*fl−*G lacking any preferential orientation in the NPs. The formation of Cu_2_O in Cu_2_O/*fl−*G is, therefore, analogous to 
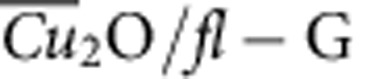
. Note that some core of metallic Cu can remain in both oriented 
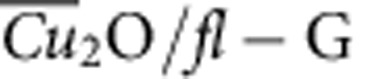
 and unoriented Cu_2_O/*fl−*G samples. Besides this Cu_2_O/*fl−*G at two different loadings, a third Cu_2_O/*fl−*G sample was prepared by adsorbing commercial Cu_2_O NPs on *fl−*G at 0.1% loading (commercial Cu_2_O/*fl−*G). Cu_2_O/*fl−*G and commercial Cu_2_O/*fl−*G are the analogous catalysts to determine the influence of particle orientation.

The Ullmann-type self coupling of iodobenzene was carried out at a Cu-to-substrate mol ratio of 2.24 × 10^−6^ mol% for facet-oriented 
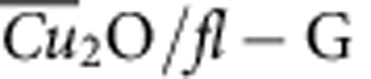
 catalyst and for the three Cu_2_O/*fl−*G analogues. Product formation at this low Cu/substrate mol ratio was only observed for 
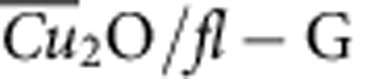
. The results obtained are presented in Table 1.

Control experiments in the absence of any Cu or G under optimized conditions using KOCH_3_ as base did not afford any product and the use as catalyst of *fl−*G in 1,4-dioxane and KOCH_3_ as base barely afforded a minimal conversion (Table 1, entry 1). In contrast, the use of oriented 
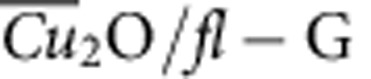
 films as catalyst and KOCH_3_ as base in 1,4-dioxane afforded a substantial conversion of 26.4%, leading to a mixture of biphenyl (**2**) and *o*- and *p*-iodobiphenyl (**3a**,**b**). The use of dimethylsulphoxide as solvent and K_2_CO_3_, KOH or KOCH_3_ as base was unsatisfactory resulting in no conversion (K_2_CO_3_ and KOH) or **1** was converted in a small percentage (KOCH_3_) without forming the expected **2**. Importantly, when Cu_2_O/*fl−*G at 1 or 0.1 wt% loadings or commercial Cu_2_O/*fl−*G containing unoriented Cu_2_O NPs were used as catalyst at a Cu-to-substrate mol ratio of 1.8 × 10^−4^ mol%, conversion of **1** was minimal or zero. XRD of commercial Cu_2_O/*fl−*G that was prepared by supporting commercial Cu_2_O in ethanol on *fl−*G is provided in Supplementary Fig. 4. When the Ullmann coupling of **1** was carried out with a Cu-to**-1** mol ratio of 7.85 × 10^−2^ mol%, then, a distribution of products similar to that obtained for *fl*−G in the absence of Cu (Table 1, entry 4) was observed. When the catalytic activity of Cu_2_O/*fl−*G with 0.1 wt% Cu_2_O loading was tested at 7.85 × 10^−3^ Cu-to-**1** mol%, then, conversion of **1** was also significantly decreased (Table 1, entry 5). All these catalytic data show that oriented 
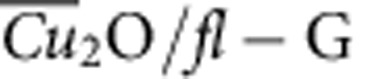
 is active at much lower Cu content than other analogous unoriented 
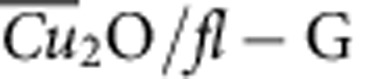
 catalysts.

Importantly, a turnover number (TON) value for iodobenzene conversion using oriented 
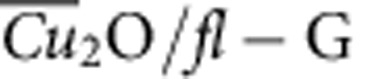
 film of 1.45 × 10^5^ was estimated for the reaction with a minimal turnover frequency (TOF) value calculated at final reaction time of 6,100 h^−1^. These numbers are much higher than those determined for conventional, randomly oriented Cu_2_O/*fl−*G or commercial Cu_2_O/*fl−*G catalysts under the same conditions. For Cu_2_O/*fl−*G (1 wt% Cu_2_O loading) the estimated TON and TOF values were 75 and 3.6 h^−1^, respectively.

During the course of the reaction, it was observed that 
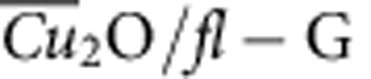
 film detaches from the quartz substrate and appears as a self-standing film in the liquid phase. Accordingly, Raman spectroscopy after the reaction reveals that although G is still present on the quartz plate, the characteristic Raman peaks associated to the presence of Cu are absent in the quartz. This detachment probably reflects exfoliation of *fl−*G during the course of the reaction. However, if the detached 
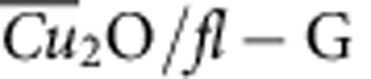
 film is recovered (now released from the quartz plates), it has been possible to perform a second use as catalyst of the 
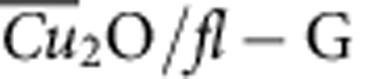
 film, reaching essentially similar values for **1** conversion and product distribution (Table 1, entry 3).

**Figure 9:**
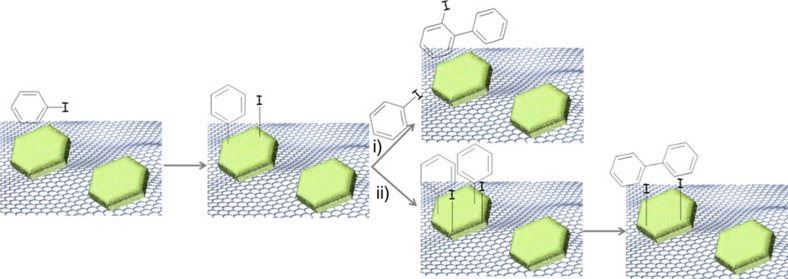
Mechanistic proposal. Reasonable reaction mechanism for the Ullmann-like self coupling of iodobenzene to form compounds **2** and **3**. Both product would have in common the Ph–Cu intermediate on the surface of Cu_2_O NP, reacting with a molecule of iodobenzene in the liquid phase (pathway *i*) or coupling with another Ph–Cu in the neighbourhood (pathway *ii*).

A plausible reaction mechanism for this iodobenzene coupling based on the knowledge of the presence of Cu(I) is proposed in Fig. 9. According to this proposal oxidative insertion of Cu(I) ions on the C–I bond will give rise to a phenyl copper and copper iodide species on the surface of Cu_2_O nanoplatelets. Phenyl copper could undergo homocoupling with a similar intermediate present of the surface of Cu_2_O NP to form **2** or, alternatively, may attack iodobenzene in the liquid phase forming positional isomers **3**. The role of the base should be the removal of I^−^ ions from the surface of Cu_2_O NPs. An important issue that remains open is the potential role of the electrical conductivity in the catalytic activity. In this regard, it is worth noting that the electrical conductivity of *fl*−G on quartz is 82 Ω□ (ref. 34) and this conductivity is not much affected by the presence of oriented Cu or Cu_2_O.

To further demonstrate the superior catalytic activity as consequence of the preferential crystal orientation, 
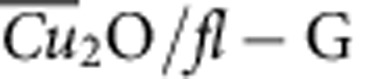
 films were also tested as catalyst for another typical Cu(I) catalytic reaction, namely the dehydrogenative silylation of n-butanol (see Eq. 2 in Table 1)^54^,^55^. It has been recently reported that G supported Cu nanoplatelets catalyze this dehydrogenative coupling reaction^14^. As in the previous case of the Ullmann-type reaction, it is of interest to determine whether or not oriented Cu_2_O NPs with preferential exposed 2.0.0 facets exhibit enhanced catalytic activity with respect to conventional randomly oriented Cu_2_O NPs supported on the same type of G. With this purpose in mind, the catalytic activity of two related samples with oriented or random NPs, namely 
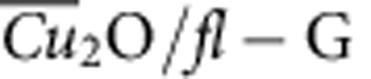
 and Cu_2_O/*fl−*G (1 or 0.1 wt% Cu_2_O loading), was compared. The dehydrogenative coupling was carried out at a Cu-to-substrate mol ratio of 2.5 × 10^−4^%.

As expected in view of the activity of Cu(I) for the dehydrogenative coupling^54^,^55^, both catalysts 
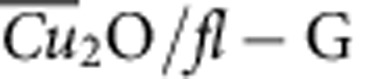
 and Cu_2_O/*fl−*G promote the reaction with almost complete selectivity towards the expected coupling product **6** at low conversion (5%). A summary of the catalytic activity data are shown in Table 1. However, as conversion increased the selectivity towards the coupling product using the 
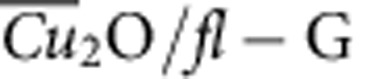
 as catalyst decreased gradually up to 60 at 34% of conversion, a fact that is not observed in the case of Cu_2_O/*fl−*G. This selectivity decrease is due to the appearance of the corresponding disiloxane, whose formation from silane **4** requires the reaction with oxygen gaining access from the ambient and this is generally observed for slow reactions (note differences in times in Table 1). As previously indicated for the Ullmann-type coupling, also in this case, detachment of the 
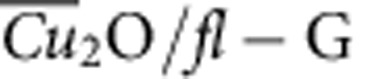
 film from the quartz plate during the course of the reaction was observed.

Control experiments using as catalysts *fl−*G or Cu_2_O NPs obtained by the polyol method adsorbed on *fl−*G at the low concentrations corresponding to the total amount of Cu present in the two 1 × 1 cm^2^ quartz plates (0.46 μg of Cu) showed no conversion of compound **4**, indicating again the high catalytic activity of oriented 
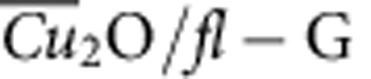
. The TOF of 
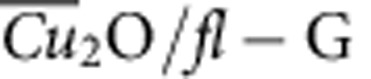
 measured at 1 h reaction time gives a value of 22,700 h^−1^ while the TOF value of Cu_2_O/*fl−*G under similar conditions was 1.4 h^−1^. No significant influence of the Cu_2_O loading on *fl−*G, either 1 or 0.1 wt%, on the TON was observed (compare entries 6 and 7 in Table 1). This remarkable difference in the TOF value is a consequence of the minute amount of Cu present on the quartz film (0.46 μg of Cu) as compared with the amount of Cu present of Cu_2_O/*fl−*G 160.000 μg, while still being able to convert **4**. We propose that this very high TOF value for 
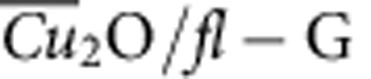
 films reflects the high catalytic activity of the material due to the preferentially exposed catalytically more active (2.0.0) facet present in 
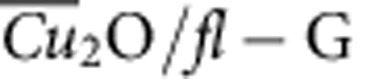
.

The third coupling reaction that was tested was C–N coupling of aniline (**7**) and bromobenzene (see Eq. 3 in Table 1). This coupling has also been reported to be promoted by Cu(I) sites in the presence of strong bases^56^,^57^. The results presented in Table 1 show that 
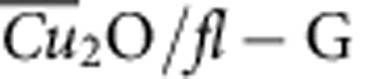
 is more efficient than Cu_2_O/*fl−*G (either at 1 or 0.1 wt% Cu_2_O loading) or commercial Cu_2_O/*fl−*G promoting C–N coupling. The estimated TON value for the disappearance of **7** using 
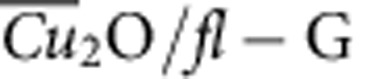
 as catalyst was 2.76 × 10^5^. The estimated TOF value at 5 h reaction time is 55,200 h^−1^. Moreover, in the last case, the high activity of 
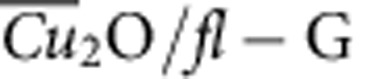
 is also reflected in the selective formation of the product of the exhaustive double C–N arylation triphenylamine (**10**). In addition, 
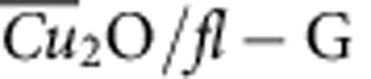
 was reusable under the reaction conditions. Although unoriented Cu_2_O/*fl−*G also exhibits activity for C–N coupling, the TON value considering **7** disappearance (TON of Cu_2_O/*fl−*G at 1 wt% Cu_2_O loading 369) is about 750 times lower than that of oriented 
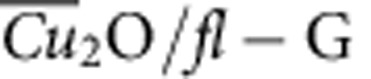
 or more than three orders of magnitude lower (TON of 74 for formation of compound **10**) if formation of product **10** is considered. Furthermore, as in the case of the Ullmann coupling, the catalytic activity of Cu_2_O/*fl−*G at 0.1 wt% Cu_2_O loading decreases almost proportionally with the diminution of the Cu_2_O content (Table 1, entry 4 in Eq. 3), resulting in no significant influence of the TON as a function of the Cu_2_O content. Overall, the results shown in Table 1 indicate again that the preferential 2.0.0 facet orientation of Cu_2_O NPs increases the catalytic activity of 
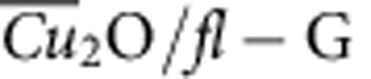
 for this reaction.

## Discussion

In the present work it has been shown that pyrolysis of Cu(NO_3_)_2_ embedded in chitosan films forms oriented 
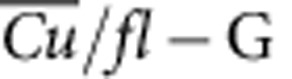
 constituted by 3 nm height Cu nanoplatelets with preferential (1.1.1) facets. We propose that this preferential growth is a result of the epitaxial templation of G on the nascent Cu nanoplatelets during phase segregation occurring at temperatures higher than 800 °C. 
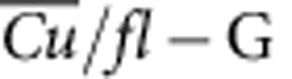
 undergoes spontaneous oxidation on exposure to the ambient to render oriented 
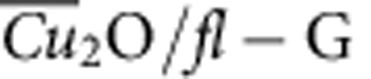
 constituted by Cu_2_O nanoplatelets with preferential 2.0.0 facets (about 82%), as determined by comparison with the raw TEM image with the image showing exclusively 2.0.0 oriented nanoplatelets. Activity data of 
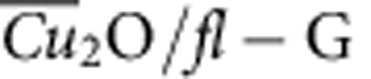
 for three typical Cu(I) catalysed couplings show that the intrinsic activity of oriented Cu_2_O nanoplatelets is about four orders of magnitude more active than analogous catalyst containing small Cu_2_O NPs on *fl−*G (1,933, 68,000 and 3,730 times for the Ullmann-like reaction, dehydrogenative silane coupling with alcohols and C–N coupling, respectively). It is proposed that this higher activity is a reflection of the intrinsic catalytic activity oriented Cu_2_O nanoplatelets. Further work in progress is aimed at showing the general scope of this procedure for the templation of other MNPs on *fl−*G. Activity data of other MNP supported on *fl−*G should allow disclosure of the specific catalytic activity of some preferred facets in oriented MNPs for other reactions.

## Methods

### Synthesis of *fl−*G

Alginic acid sodium salt from brown algae (Sigma) was pyrolysed under argon atmosphere using the following oven program: annealing at 200 °C for 2 h and, then, heating at 10 °C min^−1^ up to 900 °C for 6 h. The resulting graphitic powder was sonicated at 700 W for 1 h in water and the residue removed by centrifugation to obtain *fl−*G dispersed in water.

### Cu NPs deposition on *fl−*G

*fl−*G from alginate pyrolysis (100 mg) was added to ethylene glycol (40 ml) and the mixture was sonicated at 700 W for 1 h to obtain dispersed *fl−*G. CuCl_2_ (10.6 or 1.06 mg for the preparation of the sample at 1 or 0.1 wt% Cu_2_O) was added to the reaction mixture and Cu metal reduction was then performed at 120 °C for 24 h under continuous stirring. The Cu/*fl−*G were finally separated by filtration and washed exhaustively with water and with acetone. The resulting material was dried in a vacuum desiccator at 110 °C to remove the remaining water.

Preparation of commercial Cu_2_O/*fl−*G (0.1 wt%) was carried out by dispersing 60 mg of G from pyrolysis of alginate into 60 ml of ethanol using a ultrasound source (tip of 700 W) during 1 h and then addition of 4.5 μl of commercial ethanolic suspension of Cu_2_O (Aldrich, Ref: 678945, 1.5% (w/v)) and the mixture stirred for 12 h to achieve deposition of the nanoparticles on *fl*−G.

### Synthesis of oriented Cu NPs over *fl−*G films ( 
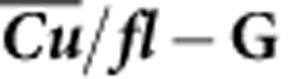
 )

Chitosan (0.5 g) from Aldrich (low molecular weight) was dissolved in a copper(II) nitrate aqueous solution (18 mg of Cu(NO_3_)_2_·2½ H_2_O in 25 ml of water). A small quantity of acetic acid (0.23 g) is necessary for complete dissolution of chitosan. The solution was filtered through syringe of 0.45 μm diameter pore to remove impurities present in commercial chitosan. The films were supported on a quartz plate (2 × 2 cm^2^) by casting 300 μl of filtered solution at 6,000 r.p.m. during 1 min. The pyrolysis was performed under argon atmosphere using the following oven program: heating rate at 5 °C min^−1^ up to 900 °C for 2 h. The amount of copper present on the films was determined by inductively coupled plasma optical emission spectrometry (ICP-OES) by immersing the plates into *aqua regia* at room temperature for 3 h and analysing the Cu content of the resulting solution.

### Ullmann-like reaction procedure

All reagents were purchased from Sigma-Aldrich and used as received without any further purification. To a solution of iodobenzene, **1** (2.0 mmol) in 4 ml of solvent (1,4-dioxane or dimethylsulphoxide) 2 mmol of base (KOCH_3_, NaOH or Na_2_CO_3_) and catalyst were added. The resulting mixture was stirred in an autoclave for 24 h at 160 °C. After the reaction, the catalyst was collected by filtration and the reaction products were analysed and identified by gas chromatography mass spectrometry (GC–MS; THERMO Electron Corporation instrument), Trace GC Ultra and DSQ, TraceGOLD: TG-5SilMS column with the following specifications: 30 m × 0.25 mm × 0.25 μm, working with a temperature program that starts at 50 °C maintained for 2 min and afterwards increasing the temperature at a rate of 10 °C min^−1^ up to 250 °C that was maintained for 10.00 min, resulting in a total run time of 32 min. The pressure of He used as the carrier gas was 0.38 Torr. Mass spectra of the products were acquired at 70,000 resolution. Biphenyl (**2**): MS (EI) *m/z* (rel.int): 154 (M^+^, 100%), 128 (4), 115 (4), 76 (12), 63 (3), 51 (3); *o*-iodobiphenyl (**3a**): MS (EI) *m/z* (rel.int): 280 (M^+^, 66.8%), 152 (100), 140 (8), 127 (8), 76 (14), 63 (4); *p*-iodobiphenyl (**3b**): MS (EI) *m/z* (rel.int): 280 (M^+^, 100%), 152(78), 140 (6), 127 (7), 76 (12), 63 (3).

### Dehydrogenative silylation reaction procedure

The catalyst 
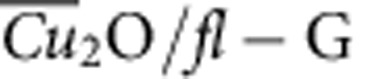
 (2 plates of 1 × 1 cm^2^, load 2.5 × 10^−4^ mol% Cu versus substrate) prepared no longer than 2 days before the reaction and stored at the ambient was introduced into a 5 ml reinforced glass reactor equipped with a magnetic bar. Then, *n-*butanol was added (10.9 mmol) under dry N_2_ atmosphere and the system purged for 15 min with N_2_. Finally, **4** (3.27 mmol) was introduced into the reactor with a syringe. The reaction mixture was stirred at 110 °C in an oil bath. At the final time, the system was allowed to cool to room temperature. The reaction was carried out in triplicate using three 
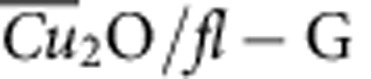
 samples prepared independently, obtaining consistent results with a deviation <8%.

In the case of powdered, unoriented Cu_2_O/*fl−*G, the catalyst was added in a 5 ml reinforced glass reactor equipped with a magnetic bar. The reactor was purged by N_2_ and the alcohol was added (10 mmol). Under inert atmosphere of N_2_ the catalyst was sonicated for a 1 h and, then, introduced in a preheated oil bath. Finally, dimethylphenylsilane **4** was introduced in the reactor with a syringe. The reaction was stirred at 110 °C. At the final reaction time the reaction mixture was allowed to cool to room temperature and the catalyst removed by filtration. In both cases (plates and powders), a known amount of *n-*dodecane was added as internal standard and the reaction was followed by monitoring periodically the reaction mixture by GC. ^1^H-NMR and MS spectra and analytical (GC retention time) data of dimethylphenylbutoxysilane were coincident with those reported in the literature^14^.

### General procedure for the C–N coupling

To a solution of bromobenzene (1.2 mmol) and aniline (1 mmol) in 4 ml of 1,4-dioxane, potassium tert-butoxide (2.1 mmol) was added as base and two pieces of 1 × 1 cm^2^ of 
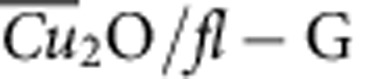
 on quartz (0.24 μg of Cu total) or unoriented Cu_2_O/*fl−*G as powder (10 mg for solid catalyst containing 1 or 0.1 wt% Cu). The resulting mixture was submitted to mechanical stirring in an autoclave for 24 h at 200 °C. The products were analysed and identified by using GC–MS (THERMO Electron Corporation instrument).

### Physicochemical characterization

Powder XRD patterns were recorded on a Schimadzu XRD-7000 diffractometer using Cu Kα radiation (*λ*=1.5418 Å, 40 kV, 40 mA) at a scanning speed of 0.20° per min in the 10–80° 2Θ range for the *ex situ* experiments. The *in situ* experiments were carried out in the 6–60° 2Θ range, with a 10 °C min^−1^ heating rate, and a flow of hydrogen or air of 10 ml min^−1^. On each plateau, the temperature was kept for 30 min before the acquisition of the diffractogram.

Raman spectra were collected with a Horiba Jobin Yvon–Labram HR UV–Visible–NIR (200–1,600 nm) Raman Microscope Spectrometer, using a laser with the wavelength of 632 nm. The spectra were collected from 10 scans at a resolution of 2 cm^−1^.

TEM images of an oriented 
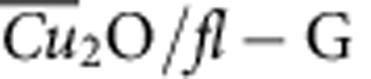
 sample were recorded at the Electron Microscopy Center of the Universitat de Valencia after abrasion of the quartz support by consecutive treatments consisting in mechanical polishing from the backside of the substrate until ∼100 μm thickness, followed by backside dimpling with a dimple grinder GATAN Model 656 and final low-angle, ion milling using an argon gun and plishing system Fishione Model 1010. Ref. 44^44^ provides the fundamentals and detailed description of the methodology.

## Additional information

**How to cite this article:** Primo, A. *et al.* High catalytic activity of oriented 2.0.0 copper(I) oxide grown on graphene film. *Nat. Commun.* 6:8561 doi: 10.1038/ncomms9561 (2015).

## Supplementary Material

Supplementary Information Supplementary Figures 1-4

## Figures and Tables

**Table 1:**
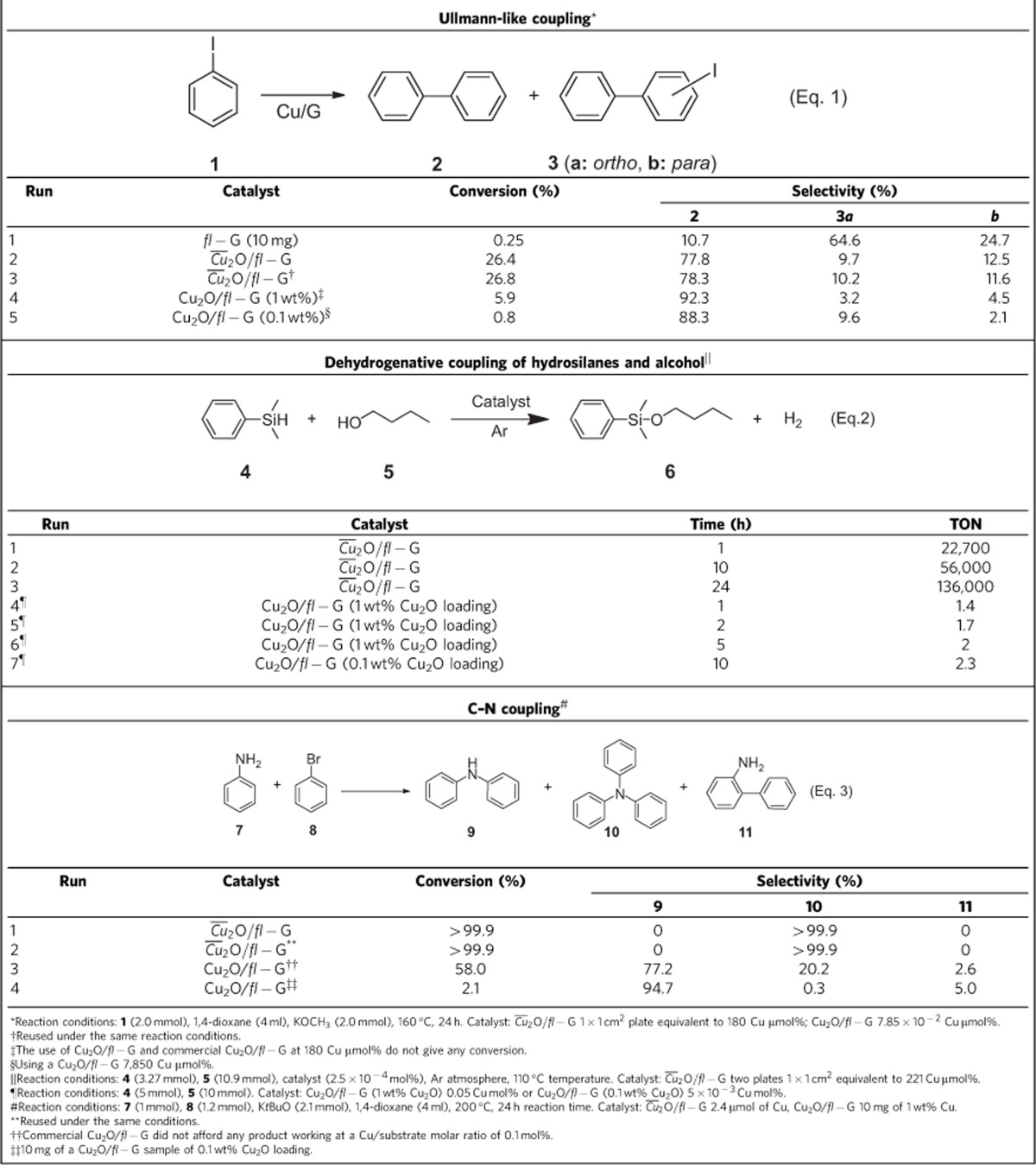
Catalytic activity of 
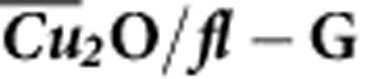
 and Cu_2_O/*fl−*G for three selected coupling reactions.
