# Supplementary material for: High catalytic activity of oriented 2.0.0 copper(I) oxide grown on graphene film
Source: Nat Commun. 2015 Oct 16;0:8561. doi: 10.1038/ncomms9561 (PMC4634216; doi:10.1038/ncomms9561)

Supplementary Fig. 1. FESEM images of Cu^{2+}-chitosan films on quartz substrate submitted to pyrolysis at 800 (a) and 900 (b) ^{o}C

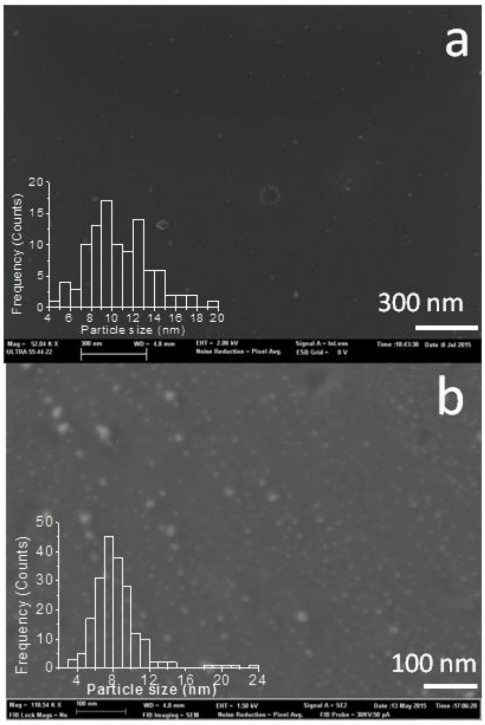

Supplementary Fig. 2. Raw TEM image recorded for $\overline{Cu}_2O/fl$-G after quartz detachment (right) and corresponding image based on electron diffraction showing those particles having preferential 2.0.0 orientation (left).

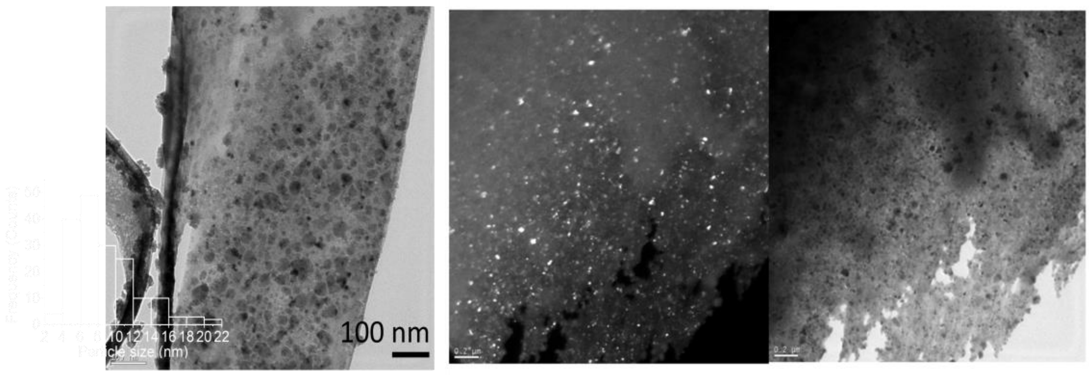

Supplementary Fig. 3. TEM images (A-C), Raman spectrum (D) and AFM data (E) of *fl*-G used for deposition of Cu NPs prepared by the polyol method.

A

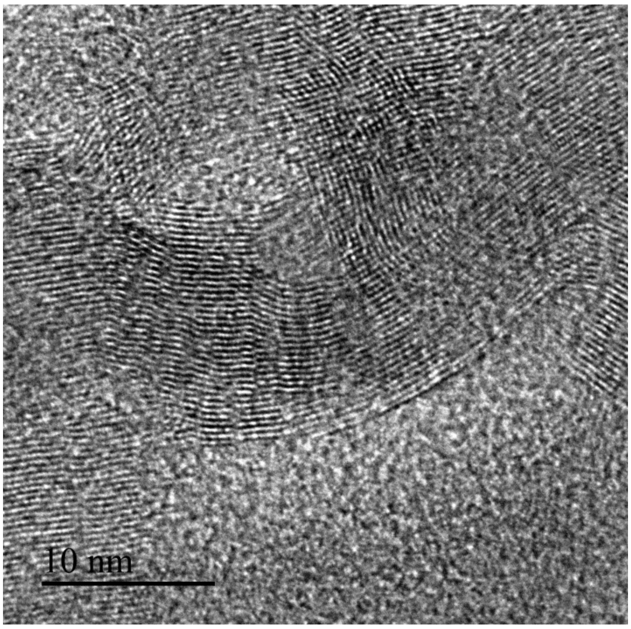

B

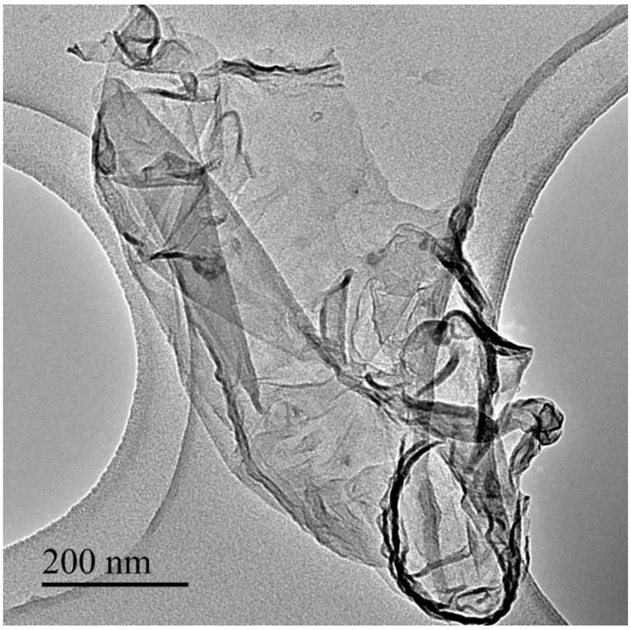

C

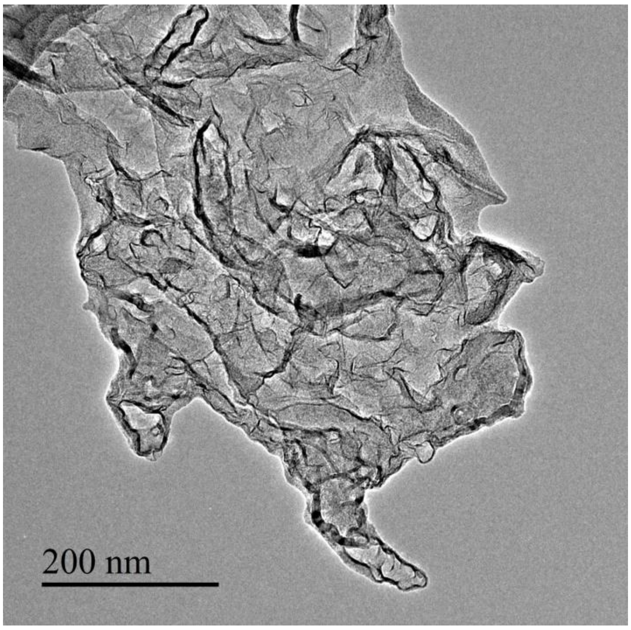
200 nm

D

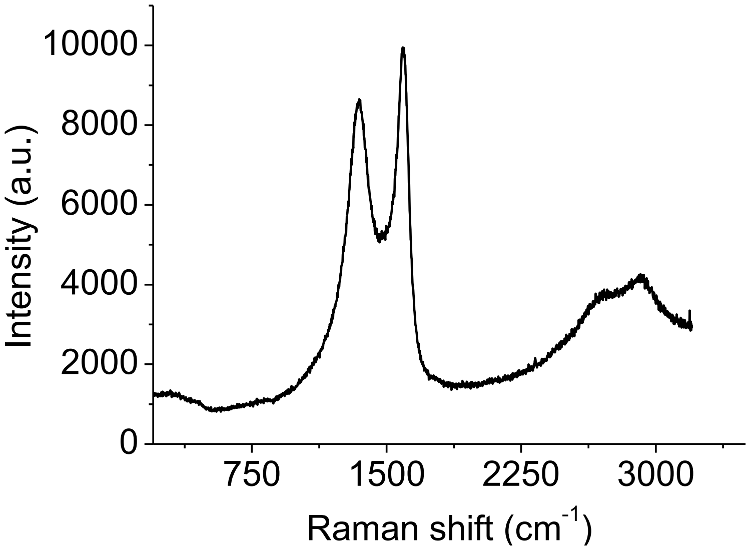
10000
8000
6000
4000
2000
0
Intensity (a.u.)
750
1500
2250
3000
Raman shift (cm-1)

F

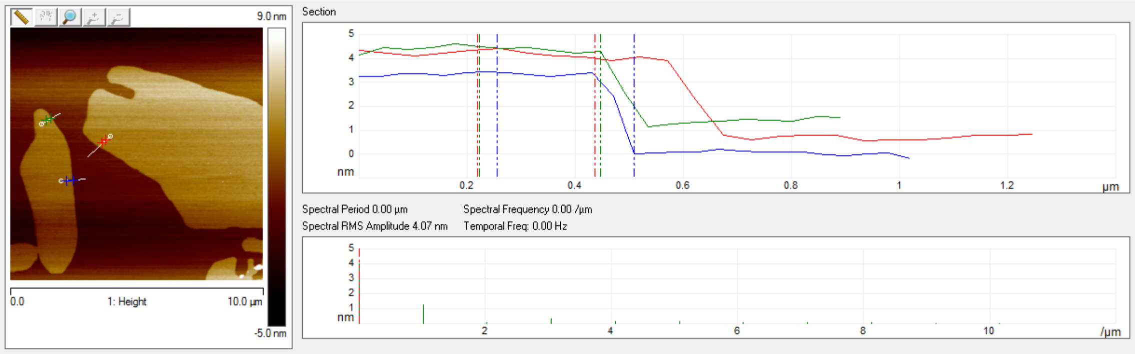

Pair	Horizontal Distance	Vertical Distance	Surface Distance	Angle	Rmax	Rz	Rz Count	Rms	Ra (Frequency Cutoff)	Frequency Cutoff	Radius	Radius Sigma
1	0.275 (µm)	-3.436 (nm)	0.275 (µm)	-0.715 (º)	3.003 (nm)	0.000 (nm)	0.000	0.082 (nm)	0.979 (nm)	0.000 (µm)	3.635 (µm)	0.586 (nm)
2	0.208 (µm)	-0.241 (nm)	0.208 (µm)	-0.066 (º)	0.600 (nm)	0.000 (nm)	0.000	0.093 (nm)	0.534 (nm)	0.000 (µm)	6.824 (µm)	0.326 (nm)
3	0.224 (µm)	-0.203 (nm)	0.224 (µm)	-0.052 (º)	0.849 (nm)	0.000 (nm)	0.000	0.149 (nm)	0.827 (nm)	0.000 (µm)	22.492 (µm)	0.101 (nm)

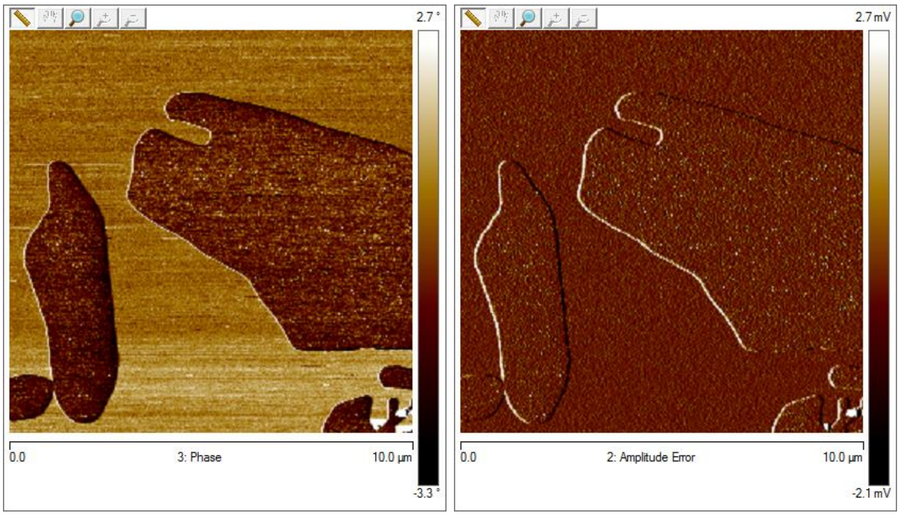

Supplementary Fig. 4. XRD pattern of commercial Cu2O nanoparticles supported on *fl*-G. The average particle size of Cu_2O determined by the Scherer equation of the most intense XRD peak is about 200 nm. Further details of the commercial Cu2O can be found in: Zhang, J., Liu, J., Peng, Q., Wang, X., & Li, Y., Nearly Monodisperse Cu2O and CuO Nanospheres: Preparation and Applications for Sensitive Gas Sensors. *Chemistry of Materials* 18, 867-871 (2006).

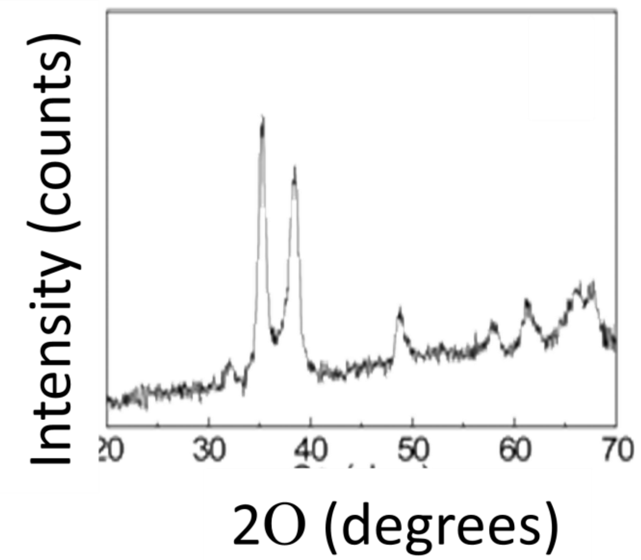

Supplement: Supplementary Information — Supplementary Figures 1-4 [file ncomms9561-s1.pdf]
